# Bioactivity Potential of *Prunus spinosa* L. Flower Extracts: Phytochemical Profiling, Cellular Safety, Pro-inflammatory Enzymes Inhibition and Protective Effects Against Oxidative Stress *In Vitro*

**DOI:** 10.3389/fphar.2017.00680

**Published:** 2017-10-11

**Authors:** Anna Marchelak, Aleksandra Owczarek, Magdalena Matczak, Adam Pawlak, Joanna Kolodziejczyk-Czepas, Pawel Nowak, Monika A. Olszewska

**Affiliations:** ^1^Department of Pharmacognosy, Faculty of Pharmacy, Medical University of Lodz, Lodz, Poland; ^2^Department of General Biochemistry, Faculty of Biology and Environmental Protection, University of Lodz, Lodz, Poland

**Keywords:** *Prunus spinosa*, oxidative stress, antioxidants, human plasma, lipoxygenase, hyaluronidase, polyphenols, LC-MS

## Abstract

Flower extracts of *Prunus spinosa* L. (blackthorn)—a traditional medicinal plant of Central and Eastern Europe indicated for the treatment of urinary tract disorders, inflammation, and adjunctive therapy of cardiovascular diseases—were evaluated in terms of chemical composition, antioxidant activity, potential anti-inflammatory effects, and cellular safety in function of fractionated extraction. The UHPLC-PDA-ESI-MS^3^ fingerprinting led to full or partial identification of 57 marker constituents (36 new for the flowers), mostly flavonoids, A-type proanthocyanidins, and phenolic acids, and provided the basis for authentication and standardization of the flower extracts. With the contents up to 584.07 mg/g dry weight (dw), 490.63, 109.43, and 66.77 mg/g dw of total phenolics (TPC), flavonoids, proanthocyanidins, and phenolic acids, respectively, the extracts were proven to be rich sources of polyphenols. In chemical *in vitro* tests of antioxidant (DPPH, FRAP, TBARS) and enzyme (lipoxygenase and hyaluronidase) inhibitory activity, the extracts effects were profound, dose-, phenolic-, and extraction solvent-dependent. Moreover, at *in vivo*-relevant levels (1–5 μg/mL) the extracts effectively protected the human plasma components against peroxynitrite-induced damage (reduced the levels of oxidative stress biomarkers: 3-nitrotyrosine, lipid hydroperoxides, and thiobarbituric acid-reactive substances) and enhanced the total antioxidant status of plasma. The effects observed in biological models were in general dose- and TPC-dependent; only for protein nitration the relationships were not significant. Furthermore, in cytotoxicity tests, the extracts did not affect the viability of human peripheral blood mononuclear cells (PBMC), and might be regarded as safe. Among extracts, the defatted methanol-water (7:3, v/v) extract and its diethyl ether and ethyl acetate fractions appear to be the most advantageous for biological applications. As compared to the positive controls, activity of the extracts was favorable, which might be attributed to some synergic effects of their constituents. In conclusion, this research proves that the antioxidant and enzyme inhibitory capacity of phenolic fractions should be counted as one of the mechanisms behind the activity of the flowers reported by traditional medicine and demonstrates the potential of the extracts as alternative ingredients for functional products supporting the treatment of oxidative stress-related pathologies cross-linked with inflammatory changes, especially in cardiovascular protection.

## Introduction

Medicinal plants as primary sources of natural bioactive compounds are attracting growing interest as constituents of functional products active in prevention and adjunctive therapy of numerous chronic diseases, including cardiovascular disorders (CVD), the leading cause of mortality in the world today. In the search of new candidates for closer investigation, ethnobotanical knowledge constitutes an important guideline indicating species with the relevant activity (Franz et al., 2011).

One of such plants might be *Prunus spinosa* L. (blackthorn or sloe)—a wild plume tree native to Europe, western Asia, north-western Africa, and naturalized in New Zealand and North America (Tutin et al., 1968). In European tradition it has been known for over 7,000 years, at first as a source of edible fruit and then also as a medicinal plant (Poonam et al., 2011; Zohary et al., 2012), used i.a. in the treatment of various circulatory system disorders. For medicinal applications the plant has been used throughout Europe with the flowers being the most popular in central and eastern parts of the continent (Hoppe, 1981). Ethnopharmacological sources indicate vasoprotective, anti-inflammatory, diuretic, detoxifying (blood purifying), and spasmolytic activities for the flowers, and document their use as ingredients of compound herbal prescriptions traditionally applied, e.g., to treat intestinal and respiratory tract disorders, but also various cardiac complaints, such as myocarditis, cardiac neurosis and atherosclerosis (Berger, 1949; Hoppe, 1981; Wawrzyniak, 1992; Blumenthal and Busse, 1998). The fruits, according to German Commission E, have been indicated mainly in mild inflammation of the oral and pharyngeal mucosa, as well as an astringent (Blumenthal and Busse, 1998); however, local European sources report their use also as a heart-strengthening remedy (Kültür, 2007; Jarić et al., 2015). Branches, on the other hand, have been more popular in the south of Europe and suggested to possess anti-hypertensive properties (Calvo and Cavero, 2014).

Active components of the plant are believed to be polyphenols, including flavonoids, A-type proanthocyanidins, anthocyanins, coumarins, and phenolic acids, forming unique and diversified profiles in particular organs, among which the flowers are the least characterized (Kolodziej et al., 1991; Sakar and Kolodziej, 1993; Olszewska and Wolbiś, 2001, 2002a,b; Guimarães et al., 2013; Pinacho et al., 2015; Owczarek et al., 2017). Some blackthorn constituents, such as flavonoid pentosides (arabinosides, xylosides, rhamnosides) and A-type procyanidin dimers with twice-bonded structures are quite rare in nature and their distribution is generally limited to selected species and plant families (Pinacho et al., 2015). This unique composition may correspond to the distinctive activity profile of *P. spinosa* reported by traditional medicine. Indeed, the earliest studies suggested that the flavonoid fraction of blackthorn flowers significantly reduces capillary permeability and shows anti-inflammatory effects in animal internal organs, normalizes the blood cholesterol and cholesterol/phospholipid ratio in atherogenic rabbits, exhibits spasmolytic effects on isolated intestinal segments from different animals, and increases the amplitude of heart contractions in perfusion of isolated frog hearts (Lisevitskaya et al., 1968; Makarov, 1968, 1972; Makarov and Khadzhaǐ, 1969). However, despite these promising *in vivo* and *ex vivo* results, the potential of blackthorn, especially the blackthorn flowers, as a source of biologically active extracts (that means standardized dry extracts—more effective than unprocessed plant materials and recommended in modern phytotherapy) remains unexplored, and the plant materials are still used mostly in the form of traditional herbal teas, partly due to the lacking molecular background for their activity and safety. Recently, special attention has been given to the antioxidant activity of the extracts from the branches, leaves, and fruits, as one of the possible mechanism of action of the blackthorn polyphenols (Barros et al., 2010; Guimarães et al., 2014; Pinacho et al., 2015). Nevertheless, as these studies were based only on simple, mostly chemical tests, and did not cover the flower extracts, the subject requires more detailed investigations.

Polyphenols, including flavonoids, are specialized plant metabolites, the beneficial effects of which in CVD is commonly linked with their ability to influence two interdependent pathological processes of oxidative/nitrative stress and inflammation (Biswas, 2016). As free radical scavengers, metal chelators, inhibitors of pro-inflammatory enzymes, and modifiers of cell signaling pathways, polyphenols may protect cellular and functional elements of the circulatory system against lipid peroxidation, protein nitration, chronic inflammation, and oxidative damage to DNA, which results i.a. in vasodilatory, vasoprotective, anti-atherogenic, antithrombotic, and anti-apoptotic effects (Alissa and Ferns, 2012; Quiñones et al., 2013). Moreover, the reducing polyphenols and their metabolites can increase the total antioxidant capacity of blood plasma and thus the tolerance of body tissues against ischemic/reperfusion injuries (Pandey and Rizvi, 2009).

Therefore, the aim of this project was a comprehensive characteristic of the flowers of *P. spinosa* in a function of fractionated extraction with respect to the chemical composition and biological activity of the dry extracts obtained with solvents of different polarity. Phenolic profiles of the extracts were investigated by UHPLC-PDA-ESI-MS^3^, HPLC-PDA, and UV-spectrophotometric methods, while their biological effects were studied *in vitro* by nine complementary tests (both chemical and human plasma models) covering some of the main mechanisms of the beneficial action of polyphenols in CVD, including direct scavenging of free radicals, inhibition of pro-inflammatory enzymes, enhancement of the total antioxidant capacity of blood plasma and protection against oxidative and nitrative damage of its lipid and protein components. Moreover, the relationship between activity of the extracts and the presence of different groups of polyphenolic constituents was explored statistically. Additionally, cellular safety of the extracts was evaluated *in vitro* in cytotoxicity tests against human peripheral blood mononuclear cells (PBMCs).

## Materials and methods

### General

HPLC grade reagents and standards, such as 2,2-diphenyl-1-picrylhydrazyl(DPPH); 2,2′-azobis-(2-amidinopropane) dihydrochloride (AAPH); 2,4,6-tris-(2-pyridyl)-*s*-triazine (TPTZ); (±)-6-hydroxy-2,2,7,8-tetramethylchroman-2-carboxylic acid (Trolox®); gallic acid monohydrate; chlorogenic acid hemihydrate; caffeic acid; kaempferol; quercetin trihydrate; isorhamnetin; rutin trihydrate; cyanidin chloride; indomethacin; bovine testis hyaluronidase; lipoxygenase from soybean; linoleic acid; 2-thiobarbituric acid; were purchased from Sigma-Aldrich (Seelze, Germany/St. Louis, MO, USA), as were analytical grade butylated hydroxyanisole (BHA); 2,6-di-*tert*-butyl-4-methylphenol (BHT); Tween® 40; xylenol orange disodium salt; and Histopaque®-1077 medium. HPLC grade solvents (acetonitrile and methanol) used for UHPLC and HPLC analyses were obtained from Avantor Performance Materials (Gliwice, Poland). A (Ca^2+^)-free phosphate buffered saline (PBS) was purchased from Biomed (Lublin, Poland). Peroxynitrite was synthesized according to Pryor et al. (1995). All immunoreagents for 3-nitrotyrosine detection were purchased from Abcam (Cambridge, UK). Pierce BCA Protein Assay Kit was obtained from Thermo Scientific (Waltham, MA, USA). All other chemicals and solvents were of analytical grade and obtained from Avantor (Poland). In all analyses redistilled water was used. For chemical tests samples were incubated in a constant temperature using a BD 23 incubator (Binder, Tuttlingen, Germany) and measured using a UV-1601 Rayleigh spectrophotometer (Beijing, China), in 10 mm quartz cuvettes. Activity tests in blood plasma models and enzyme inhibitory assays were done using 96-well plates and a microplate reader, SPECTROStar Nano (BMG LabTech, Ortenberg, Germany).

### Plant material and extracts preparation

Commercial samples of *Prunus spinosa* L. flowers were purchased in 2015 (harvest in April 2015) from Dary Natury (Koryciny, Poland). According to the manufacturer, the raw material was collected in the district Rudka, Malopolska province (50°02′N, 20°52′E). The raw material was powdered with an electric grinder, and sieved through a 0.315-mm sieve. A portion (100 g) was extracted with chloroform (3 L, 30 h) in a Soxhlet apparatus and the pellet was next four times refluxed with methanol-water (7:3, v/v; 4 × 1 L) to give the defatted methanol extract (MED, 27.3 g dw). The extraction solvent was selected from methanol-water mixtures of different alcohol concentration after optimization performed in terms of extracts yield and total phenolic content (results not shown). The MED (25.0 g) was suspended in water (1 L) and subjected to sequential liquid-liquid extraction with organic solvents (8 × 100 mL each) to yield diethyl ether fraction (DEF, 1.23 g dw), ethyl acetate fraction (EAF, 4.00 g dw), *n*-butanol fraction (BF, 4.86 g dw) and water residue (WR, 13.08 g dw). The organic solvent extracts were evaporated *in vacuo*, and the water-containing fractions were lyophilized using an Alpha 1–2/LD Plus freeze dryer (Christ, Osterode am Harz, Germany) before weighing. In further analyses freshly prepared solutions of the extracts in methanol-water (7:3, v/v) were used. All quantitative results were calculated per dry weight (dw) of the extracts.

### Phytochemical profiling

#### Qualitative LC-MS study

The UHPLC-PDA-ESI-MS^3^ analysis was performed on UHPLC-3000 RS system (Dionex, Dreieich, Germany) equipped with a dual low-pressure gradient pump, an autosampler, a column compartment, a diode array detector, and an AmaZon SL ion trap mass spectrometer with an ESI interface (Bruker Daltonik, Bremen, Germany). Separations were carried out on a Kinetex XB-C18 column (1.7 μm, 150 mm × 2.1 mm i.d.; Phenomenex, Torrance, CA, USA) at 25°C. The mobile phase consisted of solvent A (water/formic acid, 100:0.1, v/v), and solvent B (acetonitrile/formic acid, 100:0.1, v/v) with the elution profile as follows: 0–10 min, 6–13% B (v/v); 10–15 min, 13% B (v/v); 15–19 min, 13–15% B (v/v); 19–24 min, 15% B (v/v); 24–40 min, 15–23% B (v/v); 40–55 min, 23–40% B (v/v); 55–60 min, 40% B (v/v); 60–63 min, 40–6% B (v/v); 63-70 min, 6% B (equilibration). The flow rate was 0.3 mL/min. Before injection, sample solutions of the extracts (3.0 mg/mL) were filtered through a PTFE syringe filter (13 mm, 0.2 μm, Whatman, Pittsburgh, PA, USA). UV-Vis spectra were recorded over a range of 200–600 nm, and chromatograms were acquired at 254, 280, and 350 nm. The LC eluate was introduced directly into the ESI interface without splitting and analyzed in both negative and positive ion modes. ESI parameters: the nebulizer pressure was 40 psi; dry gas flow 9 L/min; dry temperature 300°C; and capillary voltage 4.5 kV. MS^2^ and MS^3^ fragmentations were obtained in Auto MS/MS mode for the most abundant ions at the time. Analysis was carried out using scan from m/z 200–2,200.

#### Quantitative standardization

The total phenolic contents (TPC) and total proanthocyanidin contents (TPA) were quantified by the Folin-Ciocalteu and *n*-butanol-HCl methods, respectively, as described previously (Olszewska et al., 2012). Results were expressed as equivalents of gallic acid (GAE) and cyanidin chloride (CYE), respectively. The total flavonoid contents (TFA) were determined by HPLC-PDA as the total content of flavonoid aglycones after acid hydrolysis. Samples of the extracts (1–5 mg) were heated under reflux for 30 min with methanol-water (9:1, v/v; 30 mL) and 25% (w/v) hydrochloric acid (9 mL). The hydrolysates were diluted with methanol-water (7:3, v/v) to 50 mL, filtered through a PTFE syringe filter (as above) and injected (5 μL) into the HPLC system. The HPLC-PDA assays were carried out according to Olszewska (2012) with quercetin, kaempferol and isorhamnetin used for external calibration. Results were re-calculated for total contents of glycosides with the molecular masses of avicularin, juglanin, and isorhamnetin diglucoside, correspondingly. The total contents of phenolic acids (TAC) were assayed by HPLC-PDA according to Olszewska et al. (2012) with caffeic acid and chlorogenic acid used as calibration standards for quantification of simple hydroxycinnamic acids and quinic acid pseudodepsides, respectively.

### Antioxidant activity in chemical models

The DPPH free-radical scavenging activity was determined according to the method optimized previously (Olszewska et al., 2012) and expressed as normalized EC_50_ values calculated from concentration-inhibition curves. The FRAP (Ferric Reducing Antioxidant Power) was determined according to Olszewska et al. (2012) and expressed in μmol of ferrous ions (Fe^2+^) produced by 1 g of the dry extract or standard, which was calculated from the calibration curve of ferrous sulfate. The ability of the extracts to inhibit AAPH-induced peroxidation of linoleic acid (LA) was assayed as described previously (Olszewska et al., 2012) with peroxidation monitored by quantification of thiobarbituric acid-reactive substances (TBARS) according to the method of Kljak and Grbeša (2015) with some changes. Briefly, before and after the samples were incubated for 3 h at 50.0 ± 0.1°C in the dark, the reaction solution (0.5 mL) was mixed with 0.67% (w/w) aqueous thiobarbituric acid (1 mL), 0.05 M HCl (0.5 mL), Tween®40 (1 mL), and then heated for 30 min in the water bath at 95°C. The absorbance was measured at 535 nm vs. the control with methanol-water (7:3, v/v) instead of the reaction mixture. The inhibition ratio (I%) of the LA-peroxidation was calculated as follows: I% = (ΔA_control_ − ΔA_sample_)/ΔA_control_, where ΔA is the difference between the absorbance measured before and after incubation. Finally, antioxidant activity was expressed as IC_50_ values calculated from concentration-inhibition curves.

### Antioxidant activity in human plasma models

#### Isolation of blood plasma and preparation of samples

Blood from five healthy, non-smoking volunteers was obtained from the Regional Centre of Blood Donation and Blood Treatment in Lodz (Poland), collected on CPD (citrate/phosphate/dextrose) solution in the Fresenius-Kabi Compoflex bags, and next plasma was isolated by differential centrifugation of blood (Kolodziejczyk-Czepas et al., 2013). All experiments were approved by the committee on the Ethics of Research at the University of Lodz 8/KBBN-UŁ/II/2015. For the FRAP assay and measurements of 3-nitrotyrosine, plasma samples were diluted with a (Ca^2+^)-free PBS buffer (1:4, v/v), whereas for hydroperoxide and TBARS assays plasma was diluted with (Ca^2+^)-free PBS in a volume ratio 1:1. For all tests, samples were pre-incubated for 5 min at 37°C with the examined extracts added to the final concentration range of 1–50 μg/mL, and then exposed to 100 μM peroxynitrite (ONOO^−^). Control samples were prepared with plasma untreated with the extracts and/or peroxynitrite. In the experiments with blood plasma and the extracts only (without adding ONOO^−^) no pro-oxidative effect was found. Protein concentration in blood plasma was estimated using bicinchoninic acid (BCA) assay (according to protocol provided by the manufacturer).

#### Determination of 3-nitrotyrosine in human plasma proteins

Detection of nitrotyrosine-containing proteins by the competitive ELISA (C-ELISA) method in plasma samples (control or antioxidants and ONOO^−^-treated plasma) was performed according to Kolodziejczyk-Czepas et al. (2013). The concentrations of nitrated proteins were estimated from the standard curve and are expressed as the 3NT-Fg equivalents (in nmol/mg of plasma protein).

#### Ferric-xylenol orange hydroperoxide assay

Concentration of hydroperoxides in plasma samples (control or antioxidants and ONOO^−^-treated plasma) was determined by a ferric-xylenol orange (FOX-1) protocol (Kolodziejczyk-Czepas et al., 2013). The FOX-1 reagent contained 125 μM xylenol orange and 100 mM sorbitol in 25 mM sulfuric acid, and was freshly prepared each time before use by the addition of ammonium ferrous sulfate to the final concentration of 250 μM. To perform FOX-1 assay, blood plasma samples were mixed with the reagent in a volume ratio 1:9 and incubated for 30 min in the dark (25°C). Absorbance of the sample was measured at 560 nm against blank (water instead of plasma). The amount of lipid hydroperoxides was calculated from the standard curve of hydrogen peroxide and expressed in nmol/mg of plasma proteins.

#### TBARS test

Determination of thiobarbituric acid reactive substances (TBARS) in plasma samples (control or antioxidants and ONOO^−^-treated plasma) was performed according to Kolodziejczyk et al. (2011). Results were expressed in μmol TBARS/mL of plasma.

#### Ferric reducing ability of plasma (FRAP) assay

The influence of the extracts on the total antioxidant activity of plasma (dependent on non-enzymatic antioxidants) was determined according to Kolodziejczyk-Czepas et al. (2014) with some modifications. The freshly collected plasma samples were diluted with a (Ca^2+^)-free PBS buffer in a volume ratio of 1:4, prepared as described above, and then added to the reagent mixture in a volume ratio of 1:10:1:1 for plasma, acetate buffer (300 mM, pH 3.6), TPTZ (10 mM, in 0.04 M hydrochloric acid), and ferric chloride (20 mM), respectively. After incubation for 15 min at 37°C, the measured FRAPs of plasma samples (control or antioxidants and ONOO^−^-treated plasma) were expressed in mM Fe^2+^ in plasma.

### Inhibition of pro-inflammatory enzymes

The ability of the extracts to inhibit lipoxygenase (LOX) and hyaluronidase (HYAL) was examined as described by Michel et al. (2017) with some changes. Briefly, in the LOX tests the reagents were used in a volume ratio of 1:2:1 for working solutions of the tested analyte, linoleic acid, and enzyme. Results of the both assays were expressed as IC_50_ values calculated from concentration-inhibition curves.

### Cell viability assay

PBMCs were isolated from human blood (obtained and collected as described above) using the Histopaque®-1077 medium (a sterile solution of polysucrose, 57 g/L, and sodium diatrizoate, 90 g/L, with a density of 1.077 g/mL). From each of eight donors, two independent PBMCs isolations and incubations with the extracts were performed. Blood was carefully layered (in a volume ratio of 1:1) onto the medium, and centrifuged for 30 min (400 × g, at room temperature). Then, the pellet was washed two times with 0.02 M PBS buffer. The obtained fraction of PBMCs was suspended in PBS. Cell suspensions (1 × 10^6^ PBMCs/mL) were incubated with plant extracts, added to the final concentration of 5 μg/mL. Cell viability (%) was determined during a spectrofluorimetric analysis, involving the use of propidium iodide as a fluorescent dye. Measurements were conducted using a microchip-type automatic cell counter Adam-MC DigitalBio (NanoEnTek Inc., Seoul, Korea), after 60 and 120 min of incubation of PBMCs with the examined substances (at 37°C). The procedure was carried out according to the manufacturer's protocol.

### Statistical analysis

Normality of the distribution of the results was verified using the Shapiro-Wilk test, and the homogeneity of variances using the Levene's test. The results are reported as means ± SD (standard deviation) or ± SE (standard error) for the indicated number of experiments. The significance of differences between samples and controls was determined with one-way ANOVA (for chemical tests) or one-way ANOVA for repeated measures (for human plasma model), followed by the *post-hoc* Tukey's test for multiple comparisons. The correlations were evaluated using *F*-test. All calculations were performed using the Satistica12Pl software for Windows (StatSoft Inc., Krakow, Poland) with *p*-values less than 0.05 regarded as significant.

## Results

### LC-MS metabolite profiling

**Figure 1:**
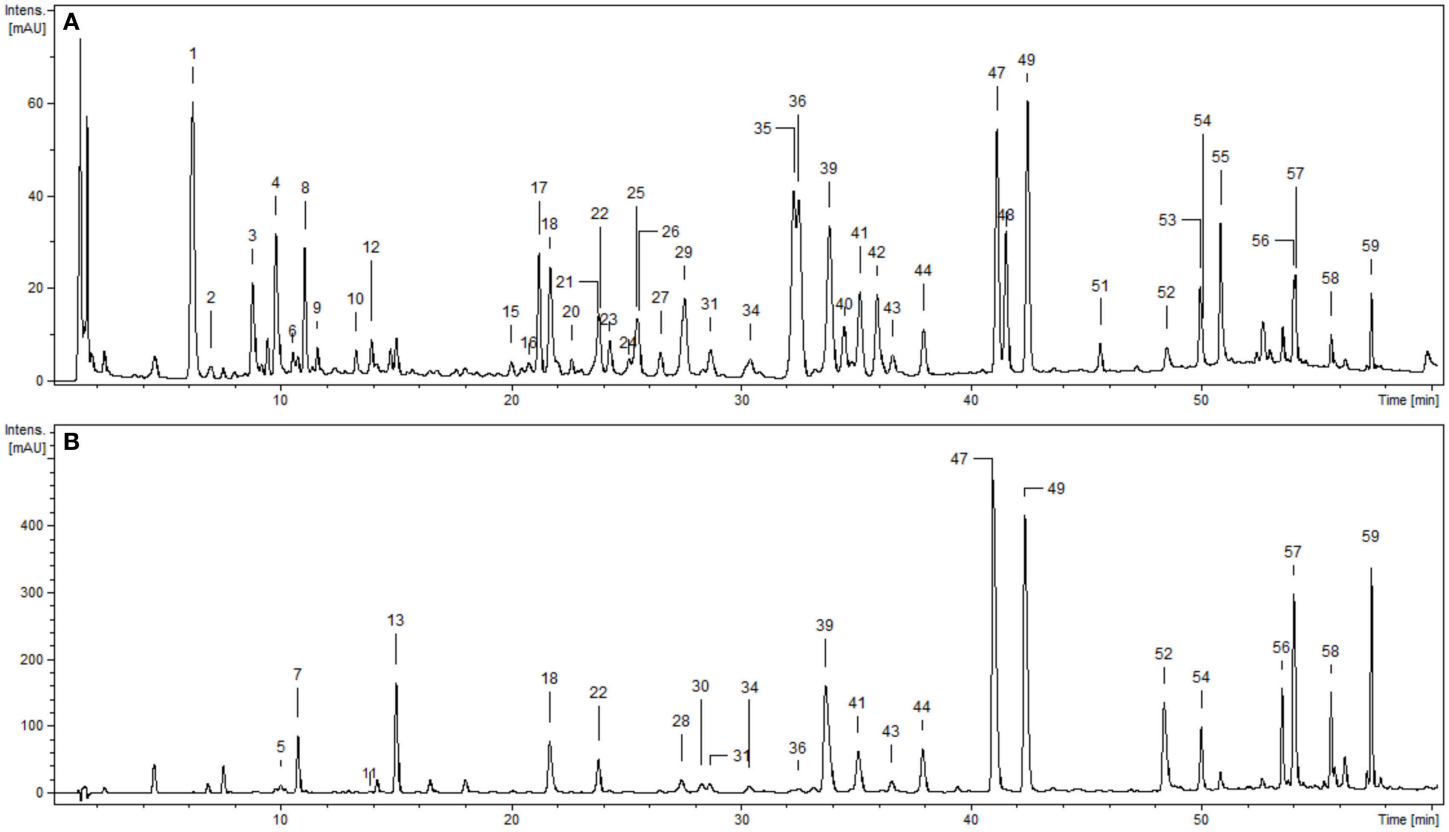
Representative UHPLC chromatograms of the *P. spinosa* flower dry extracts at 280 nm**: (A)** MED, defatted methanol-water (7:3, v/v) extract; **(B)** DEF, diethyl ether fraction. Peak numbers refer to those implemented in Table 1.

Phytochemical profiling of the dry extracts revealed significant differences in their chemical composition depending on the extraction solvent (Figure 1, Tables 1, 2). The qualitative UHPLC-PDA-ESI-MS^3^ analysis resulted in full or partial identification of over fifty phenolic constituents (Figure 1, Table 1, peaks **1**–**59**) belonging to three main classes of phytochemicals—flavonols (thirty eight analytes), flavan-3-ol derivatives (catechins and proanthocyanidins, seven peaks) and phenolic acids (twelve compounds). The analytes were structurally characterized based on the comparison of their chromatographic behavior and ESI-MS^3^ fragmentation patterns (in positive and negative ionization modes) with the literature data or reference standards, both commercial and isolated previously in our laboratory from flowers and leaves of *P. spinosa* (Olszewska and Wolbiś, 2001, 2002a,b; Owczarek et al., 2017). The greatest chemical diversity was observed for the defatted methanol-water (7:3, v/v) extract (MED), while its fractions of diethyl ether (DEF), ethyl acetate (EAF), *n*-butanol (BF), and water residue (WR) obtained after sequential liquid-liquid partitioning were enriched in selected analytes, depending on the fractionation solvent.

**Table 1:** UHPLC-PDA-ESI-MS^3^ data of polyphenols detected in *P. spinosa* flower dry extracts.

**Peak**	**Analyte**	***R_*t*_* (min)**	**UV λ_max_ (nm)**	**[M–H]^−^ (m/z)**	**Fragmentary ions**	**[M+H]^+^ (m/z)**	**Fragmentary ions**	**Formula**	**Extract**	**References**
1	3-*O*-Caffeoylquinic acid (neochlorogenic acid)^a^^,^^b^	6.2	325	353	191, 179	355	163	C_16_H_18_O_9_	BF, EAF, MED, WR	[1]
2	Caffeic acid hexoside^b^	7.0	325	341	179	365	185	C_15_H_18_O_9_	BF, MED	
3	3-*O*-*p*-Coumaroylquinic acid^b^	8.8	310	337	163, 191	339	147	C_16_H_18_O_8_	BF, EAF, MED, WR	[1]
4	5-*O*-Caffeoylquinic acid (chlorogenic acid)^a^^,^^b^	9.8	325	353	191, 179	355	163	C_16_H_18_O_9_	BF, EAF, MED, WR	[1]
5	(+)-Catechin (CA)^a^^,^^b^	10.0	280	289	245	291	139	C_15_H_14_O_6_	DEF	[5]
6	3-*O*-Feruloylquinic acid^b^	10.5	325	367	193	369	177	C_17_H_20_O_9_	BF, EAF, MED, WR	[1]
7	Caffeic acid^a^^,^^b^	10.7	325	179	–	181	–	C_9_H_8_O_4_	DEF	[5]
8	4-*O*-Caffeoylquinic acid (cryptochlorogenic acid)^a^^,^^b^	11.0	325	353	173, 191, 135	355	163	C_16_H_18_O_9_	BF, EAF, MED, WR	[1]
9	Ferulic acid hexoside^b^	11.5	325	355	193	379	217	C_16_H_20_O_9_	BF, EAF, MED	
10	5-*O*-*p*-Comaroylquinic acid^b^	13.3	310	337	191, 163	339	177	C_16_H_18_O_8_	BF, EAF, MED	[1]
11	(–)-Epicatechin^a^^,^^b^	13.7	280	289	245	291	139	C_15_H_14_O_6_	DEF	[5]
12	4-*O*-*p*-Comaroylquinic acid^b^	13.9	310	337	173, 163	339	177	C_16_H_18_O_8_	BF, EAF, MED	[1]
13	*p*-Coumaric acid^a^^,^^b^	15.1	310	163	–	165	–	C_9_H_8_O_3_	DEF	
14	4-*O*-Feruloylquinic acid^b^	15.7	325	367	173, 191	369	177	C_17_H_20_O_9_	BF	[1]
15	Kaempferol hexoside^b^	20.0	264, 355, 285	447	357, 327, 287	449	431, 329	C_21_H_20_O_11_	BF, EAF, MED	
16	(Epi)catechin-A-(epi)catechin	20.8	280	575	423, 289	577	559, 425, 287	C_30_H_24_O_12_	EAF, MED	[2], [3], [4], [5]
17	Isorhamnetin dihexoside^b^	21.1	265, 353	639	459, 315	641	479, 317	C_28_H_32_O_17_	BF, MED	
18	(Epi)catechin-A-(epi)catechin	21.7	280	575	539, 423, 289	577	559, 425, 287	C_30_H_24_O_12_	DEF, MED	[2], [3], [4], [5]
19	Kaempferol rhamnoside-hexoside^b^	22.0	260, 358	593	447, 285	595	449, 287	C_27_H_30_O_15_	BF	
20	Kaempferol dihexoside^b^	22.6	264, 355	609	447, 429, 285	611	449, 287	C_27_H_30_O_16_	BF, MED	
21	Quercetin hexoside-pentoside^b^	23.6	265, 358	595	505, 433, 301	597	435, 303	C_26_H_28_O_16_	BF, MED	
22	Unknown compound	23.7	280	433	287	435	289		EAF, DEF, MED	
23	Kaempferol 3-*O*-α-l-arabinopyranoside-7-*O*-α-l-rhamnopyranoside^a^^,^^b^	24.3	268, 356	563	417, 285	565	419, 287	C_26_H_28_O_14_	BF, MED	[5], [9]
24	Quercetin 3-*O*-β-d-galactoside (hyperoside)^a^^,^^b^	25.1	264, 355	463	301	465	303	C_21_H_20_O_12_	EAF, MED	
25	Quercetin 3-*O*-(6′′-*O*-α-l-rhamnopyranosyl)-β-d-glucopyranoside (rutin)^a^^,^^b^	25.4	265, 354	609	301	611	303	C_27_H_30_O_16_	BF, MED	[5]
26	Kaemferol 3-O-β-d-xylopyranoside-7-O-α-l-rhamnopyranoside (lepidoside)^a^^,^^b^	25.5	265, 354	563	431, 417, 285	565	433, 287	C_26_H_28_O_14_	EAF, MED	[9]
27	Quercetin 3-*O*-β-d-glucopyranoside (isoquercitrin)^a^	26.4	265, 356	463	301	465	303	C_21_H_20_O_12_	EAF, MED	[7]
28	(Epi)catechin-A-(epi)catechin	27.3	280	575	539, 449, 289	577	559, 425, 287	C_30_H_24_O_12_	EAF, DEF	[2], [3], [4], [5]
29	Quercetin 3-*O*-(2′′-*O*-β-d-glucopyranosyl)-α-l-arabinofuranoside^a^^,^^b^	27.4	258, 354	595	433, 301	597	435, 303	C_26_H_28_O_16_	BF, MED	[8]
30	(Epi)afzalechin-A-(epi)catechin	28.2	280	559	523, 407, 289	561	543, 409, 271	C_30_H_24_O_11_	EAF, DEF	[2], [3], [4], [5]
31	Quercetin 3-*O*-α-d-xylopyranoside (reinutrin)^a^	28.5	256, 356	433	301	435	303	C_20_H_18_O_11_	EAF, DEF, MED	[7]
32	Kaempferol rhamnoside-hexoside^b^	28.7	256, 356	593	447, 285	595	449, 287	C_27_H_30_O_15_	BF	
33	Kaempferol hexoside-pentoside^b^	30.2	254, 358	579	417, 285	581	419, 287	C_26_H_28_O_15_	BF	
34	Quercetin 3-*O*-α-l-arabinopyranoside (guaiaverin)^a^	30.4	255, 355	433	301	435	303	C_20_H_18_O_11_	EAF, DEF, MED	[7]
35	Kaempferol 3,7-di-*O*-α-l-rhamnopyranoside (kaempferitrin)^a^	32.1	254, 356	577	431, 285	579	433, 287	C_27_H_30_O_14_	BF, EAF, MED	[7], [9]
36	Kaempferol 3-*O*-α-l-arabinofuranoside-7-O-α-l-rhamnopyranoside^a^	32.4	254, 356	563	431, 285	565	433, 287	C_26_H_28_O_14_	BF, EAF, DEF, MED	[9]
37	(Epi)afzalechin-A-(epi)catechin	33.2	280	559	523, 407, 289	561	543, 409, 271	C_30_H_24_O_11_	EAF	[2], [3], [4], [5]
38	Kaempferol hexoside-rhamnoside^b^	33.6	255, 356	593	285	595	449, 287	C_27_H_30_O_15_	BF	
39	Quercetin 3-*O*-α-l-arabinofuranoside (avicularin)^a^	33.7	255, 355	433	301	435	303	C_20_H_18_0_11_	EAF, DEF, MED	[5], [6]
40	Quercetin 3-*O*-(4′′-*O*-β-d-glucopyranosyl)-α-l-rhamnopyranoside (multinoside A)^a^	34.3	254, 356	609	447, 301	611	449, 303	C_27_H_30_O_16_	BF, EAF, MED	[7]
41	Quercetin 3-*O*-α-l-rhamnopyranoside (quercitrin)^a^	35.1	255, 355	447	301	449	303	C_21_H_20_O_11_	EAF, DEF, MED	[7]
42	Kaempferol hexoside-pentoside^b^	36.0	254, 355	579	417, 285	581	419, 287	C_26_H_28_O_15_	BF, EAF, MED	
43	Kaempferol pentoside^b^	36.3	254, 355	417	285	419	287	C_20_H_18_O_10_	EAF, DEF, MED	
44	Kaempferol 3-*O*-β-d-xylopyranoside^a^	37.8	255, 356	417	285	419	287	C_20_H_18_O_10_	EAF, DEF, MED	[6]
45	Kaempferol hexoside^b^	39.9	255, 356	447	285	449	287	C_21_H_20_O_11_	EAF	
46	Kaempferol hexoside-rhamnoside^b^	40.4	254, 356	593	431, 285	595	433, 287	C_27_H_30_O_15_	BF	
47	Kaempferol 3-*O*-α-l-arabinofuranoside (juglanin)^a^	41.0	254, 356	417	285	419	287	C_20_H_18_O_10_	EAF, DEF, MED	[6]
48	Kaempferol 3-*O*-(4′′-*O*-β-d-glucopyranosyl)-α-l-rhamnopyranoside (multiflorin B)^a^	41.4	256, 354	593	285	595	433, 287	C_27_H_30_O_15_	BF, EAF, MED	[7]
49	Kaempferol 3-*O*-α-l-rhamnopyranoside (afzelin)^a^	42.3	256, 356	431	285	433	287	C_21_H_20_O_10_	EAF, DEF, MED	[6]
50	Quercetin 7-*O*-α-l-ramnopyranoside^b^	43.5	254, 356	447	301	449	303	C_21_H_20_O_11_	EAF	
51	Quercetin acetyl-hexoside-rhamnoside^b^	45.4	255, 356	651	609, 447, 301	653	449, 413, 303	C_29_H_32_O_17_	EAF, MED	
52	Quercetin^a^	48.3	255, 356	301	–	303	–	C_15_H_10_O_7_	EAF, DEF, MED	[5], [6]
53	Kaempferol acetyl-hexoside-rhamnoside^b^	49.8	255, 355	635	593, 285	637	619, 415, 353, 287	C_29_H_32_O_16_	EAF, MED	
54	Kaempferol 7-*O*-α-l-ramnopyranoside^a^	50.0	254, 356	431	285	433	287	C_21_H_20_O_10_	BF, EAF, DEF, MED	[6]
55	Unknown compound	50.8	310	614	452, 358, 316	616	454, 436		EAF, MED	
56	Kaempferol^a^	53.8	255, 356	285	–	287	–	C_15_H_10_O_6_	EAF, DEF, MED	[5], [6]
57	Kaempferol 3-*O*-(2′′-*O*-*E*-*p*-coumaroyl)-α-l-arabinofuranoside-7-*O*-α-l-rhamnopyranoside^a^	53.9	267, 316, 355	709	563, 285	711	565, 279	C_35_H_34_O_16_	EAF, DEF, MED	[8]
58	Quercetin *p*-coumaroyl-pentoside^b^	55.4	267, 316, 355	579	433, 301	581	279	C_29_H_24_O_13_	DEF, MED	
59	Kaempferol 3-*O*-(2′′-*O*-*E*-*p*-coumaroyl)-α-l-arabinofuranoside^a^	57.3	267, 316, 355	563	285	565	279	C_29_H_24_O_12_	EAF, DEF, MED	[6]

a *Identified with authentic standards*.

b *Detected in P. spinosa flowers for the first time. R_t_, retention times. UV λ_max_, absorbance maxima in PDA spectra. [M–H]^−^, pseudomolecular ions in MS spectra recorded in a negative mode. [M+H]^+^, pseudomolecular ions in MS spectra recorded in a positive mode. MED, defatted methanol-water (7:3, v/v) extract. DEF, diethyl-ether fraction. EAF, ethyl acetate fraction. BF, n-butanol fraction. WR, water residue. References: [1] Clifford et al. (2003); [2] Hamed et al. (2014); [3] Li and Deinzer (2008); [4] Kolodziej et al. (1991); [5] Pinacho et al. (2015); [6] Olszewska and Wolbiś (2001); [7] Olszewska and Wolbiś (2002a); [8] Olszewska and Wolbiś (2002b); [9] Owczarek et al. (2017)*.

**Table 2:** Quantitative standardization data for *P. spinosa* flower dry extracts.

**Extract**	**Total phenolics^a^ TPC (mg GAE/g dw)**	**Total flavonoids (mg/g dw)^b^**				**Total proanthocyanidins TPA (mg CYE/g dw)^c^**	**Total phenolic acids TAC (mg/g dw)^d^**
		**Kaempferol**	**Quercetin**	**Isorhamnetin**	**TFC**		
MED	206.07 ± 10.86^B^	62.47 ± 0.17^B^	19.30 ± 0.39^B^	1.47 ± 0.13^B^	125.12 ± 0.55^B^	45.13 ± 2.38^B^	29.24 ± 0.76^C^
DEF	464.57 ± 20.57^D^	259.68 ± 3.30^E^	61.14 ± 2.22^E^	5.16 ± 0.01^D^	490.63 ± 8.16^E^	49.5 ± 2.23^B^	8.76 ± 0.27^A^
EAF	584.07 ± 12.98^E^	158.69 ± 1.32^D^	53.21 ± 1.15^D^	4.57 ± 0.29^C^	325.53 ± 4.23^D^	109.43 ± 3.71^C^	17.20 ± 0.47^B^
BF	296.57 ± 3.28^C^	123.05 ± 1.99^C^	32.06 ± 1.26^C^	4.58 ± 0.03^C^	241.27 ± 4.74^C^	46.6 ± 1.14^B^	66.77 ± 2.86^D^
WR	64.6 ± 1.93^A^	0.80 ± 0.01^A^	0.38 ± 0.02^A^	0.06 ± 0.01^A^	1.88 ± 0.04^A^	12.43 ± 0.25^A^	17.71 ± 0.30^B^

*Results are presented as mean values ± SD (n = 3) calculated per dry weight (dw) of the extract. For extract codes see Table 1. Different superscripts (capitals) in each column indicate significant differences in the means at p < 0.05*.

a *Values expressed in gallic acid equivalents*.

b *Values expressed as the levels of individual aglycones released after acid hydrolysis, and TFC, total glycosides*.

c *Values expressed in cyanidine chloride equivalents*.

d *Values calculated as a sum of caffeic acid and chlorogenic acid equivalents*.

Compounds **1–4**, **6–10**, and **12–14** displayed absorption maxima at 325 or 310 nm and UV-Vis spectra characteristic of caffeic acid or *p*-coumaric acid derivatives, respectively. According to the spectral profiles and hierarchical discrimination key proposed by Clifford et al. (2003), the peaks showing parent [M–H]^−^ ions at m/z 353, 337, and 367 were identified as isomeric caffeoylquinic acids (chlorogenic acids; **1**, **4**, **8**), *p*-coumaroylquinic acids (**3, 10**, **12**), and feruloylquinic acids (**6**, **14**), correspondingly. The identity of chlorogenic acids was additionally confirmed by experiments with standards, as was the presence of simple caffeic (**7**) and *p*-coumaric acids (**13**) giving the typical [M–H]^−^ ions at m/z 179 and 163, respectively. The same ions were found in MS^2^ spectra of compounds **2** and **9** after neutral losses of sugar moieties (–162 Da each), which led to the tentative identification of hexosides of caffeic acid and ferulic acid, respectively.

Peaks **5**, **11**, **16**, **18**, **28**, **30**, and **37** with UV maxima at 280 nm were classified as flavan-3-ols and proanthocyanidins. Based on standard spiking and literature data, compounds **5** and **11** were characterized as (+)-catechin and (−)-epicatechin, respectively. Compounds **16**, **18**, and **28** with parent ions [M+H]^+^ at m/z 577 were classified as dimeric A-type procyanidins. These compounds gave secondary ions in MS^2^ spectra at m/z 559, 425, and 287. The ions at m/z 425, arising from retro-Diels-Alder (RDA) fission of the [M+H]^+^ (577–152 Da), and the ions at m/z 287 (577–290 Da) resulting from quinone methide (QM) fission, confirmed the presence of two (epi)catechin units in the structures (Li and Deinzer, 2008; Hamed et al., 2014). Compounds **16**, **18**, and **28** were thus tentatively identified as (epi)catechin-A-(epi)catechins. Two A-type proanthocyanidins of that kind have already been isolated from the flowers of *P. spinosa* and identified as *ent*-epicatechin-(4α → 8;2α → O → 7)-catechin and *ent*-epicatechin-(4α → 8;2α → O → 7)-epicatechin (Kolodziej et al., 1991). Compounds **30** and **37** with pseudomolecular [M+H]^+^ ions at m/z 561 and ions in MS^2^ spectra at m/z 543, 409 and 271 were classified as dimeric A-type proanthocyanidins. The mass difference between compounds **16**, **18**, **28**, and **30**, **37** was 16 Da, which indicated that in the latter molecules one (epi)catechin unit is probably replaced by (epi)afzelechin, the flavan-3-ol reported to occur in blackthorn flowers (Kolodziej et al., 1991) and branches (Pinacho et al., 2015). m/z 409 (561–152 Da) arising from RDA fission of the [M+H]^+^ ion, confirmed the presence of (epi)catechin in both compounds. On the other hand, the product ions at m/z 271 (561–290 Da) resulting from QM fission of the [M+H]^+^ ions, showed that the upper unit in both cases was (epi)afzelechin, and hence the terminal unit must be (epi)catechin, m/z 287 (Li and Deinzer, 2008; Hamed et al., 2014). The structures of compounds **30** and **37** were thus tentatively identified as (epi)afzelechin-A-(epi)catechin. Two proanthocyanidins of that type, i.e., *ent*-epiafzelechin-(4α → 8;2α → O → 7)-epicatechin and *ent*-epiafzelechin-(4α → 8;2α → O → 7)-catechin have previously been isolated from blackthorn flowers (Kolodziej et al., 1991).

Peaks **15**, **17**, **19–21**, **23–27**, **29**, **31–36**, **38–54**, and **56–59** with two UV-Vis maxima at 250–268 and 355–365 nm were classified as flavonoids. Compounds **52** and **56** showed parent [M–H]^−^ ions at m/z 301 and 285, respectively, and were identified with standards as free aglycones quercetin and kaempferol. Peaks **23**, **26**, **35**, **36**, **44**, **47–49**, **54**, **57**, and **59** with fragment ions in MS^2^ m/z 285 (typical for kaempferol) were identified with authentic standards of kaempferol glycosides (Table 1). Compounds **15**, **32**, **33**, **38**, **42**, **43**, **45**, and **46** were tentatively characterized based on their fragmentation pattern as kaempferol mono- and diglycosides. Peak **53** with parent [M–H]^−^ ion at m/z 635 and fragment ions at m/z 593, 285 in MS spectra was tentatively identified as kaempferol acetyl hexoside-rhamnoside due to the neutral loss of an acetyl moiety (−42 Da), hexose (−162 Da) and rhamnose (−146 Da). Peaks **24**, **25**, **27**, **31**, **34**, **39–41**, and **50** with fragment ions in MS^3^ spectra at m/z 301 (typical for quercetin) were identified with standards of quercetin glycosides (Table 1). Compound **21** with pseudomolecular [M–H]^−^ ion at m/z 595 and fragment ion at m/z 301 was tentatively assigned to quercetin hexoside-pentoside. Peak **51** with parent ion at m/z 651 and fragment ions at m/z 609, 447, and 301 was determined as a quercetin analog of **53**, i.e., quercetin acetyl hexoside-rhamnoside. Similarly, compound **58** was tentatively identified as an analog of **59**, i.e., quercetin *p*-coumaroyl-pentoside, probably quercetin 3-*O*-(2′′-*E*-*p*-coumaroyl)-α-l-arabinofuranoside, due to the additional UV-Vis absorption maximum at 316 nm (typical for an aromatic acyl unit), and the neutral loss of *p*-coumaroyl moiety (−146 Da) and a pentose (−132 Da). Peak **17** gave the parent ion at m/z 639 and its MS spectra revealed the cleavage of two hexoses (−162 amu twice) and the aglycone signal consistent with isorhamnetin, which enabled tentative identification of isorhamnetin dihexoside.

### Quantitative standardization

According to the LC-MS findings, total contents of polyphenols (TPC), flavonoids (TFC), proanthocyanidins (TPA), and phenolic acids (TAC) were selected as standardization targets (Table 2). The TPC levels assayed by the standard Folin-Ciocalteu method and expressed in gallic acid equivalents (GAE) varied in the range of 64.6–584.07 mg GAE/g dw of the extracts with the highest values found for EAF (584.07 mg GAE/g dw) and DEF (464.57 mg GAE/g dw). The total flavonoid content (TFC) was determined by HPLC-PDA as a sum of flavonoid aglycones released after acid hydrolysis of native extracts, and recalculated on the dominant glycosides. With the levels constituting 55.7–105.5% of the TPC values, flavonoids were the dominant phenolic components of all extracts except WR, in which phenolic acids prevailed. The highest TFC contents were observed for DEF (490.63 mg/g dw) and EAF (325.53 mg/g dw). As regards individual aglycones, kaempferol was the dominant one and constituted 64.4–79.7% of the total aglycones. The contents of quercetin and isorhamnetin were much lower and constituted 18.8–30.9% and 1.6–4.7% of the sum of aglycones, respectively. The total TPA, determined by the *n*-butanol-HCl assay and expressed as cyanidin chloride equivalents (CYE), ranged from 12.43 to 109.43 mg CYE/g dw, with the peak value found again for EAF. Contrastingly, the highest level of phenolic acids (TAC), assayed by HPLC-PDA, was observed for BF (66.77 mg/g dw), while in other extracts the TAC contents varied in the range of 8.76–29.24 mg/g dw.

### Antioxidative effects in chemical models

All of the extracts showed significant and dose-dependent antioxidant activity and ability to scavenge free radicals (DPPH, a stable synthetic radical), directly reduce transition metal ions (Fe^3+^, FRAP), and inhibit AAPH-induced linoleic acid peroxidation (chemical model of lipid peroxidation) significantly diminishing levels of thiobarbituric acid-reactive substances (TBARS), with the final parameters strongly influenced by the extraction solvent (Table 3). Regardless of the test, activity of the extracts decreased in similar order, i.e., EAF ≥ DEF > BF > MED > WR, and correlated with the amounts of polyphenols (TPC) and flavonoids (TFC) in the samples (Table 4). The strongest relationships were observed for the FRAP test, whereas for the DPPH and TBARS assays, the correlation coefficients were negatively influenced by WR, a virtually flavonoid-free extract. If WR was excluded from the data matrix, the impact of TPC (*|r|* > 0.91, *p* < 0.05) on the antioxidant parameters of the other extracts was evident for all tests (results not shown). In a comparison to the positive standards, the antioxidant capacity of the most active extracts EAF and DEF was higher (*p* < 0.05) or not statistically different (*p* > 0.05) than that of an industrial antioxidant BHT (DPPH and TBARS tests), and Trolox, a synthetic analog of vitamin E (FRAP and TBARS tests). Moreover, if activity parameters of the native extracts were recalculated to GAE (Table 3) using the TPC values, the obtained antioxidant capacities of the phenolic fractions constituting the dry extracts were comparable to all of the positive standards except quercetin.

**Table 3:** Antioxidant activity of *P. spinosa* flower dry extracts and standard antioxidants in DPPH, FRAP, and TBARS tests.

**Analyte**	**DPPH-Radical scavenging activity**		**Ferric reducing antioxidant power**		**LA-Peroxidation TBARS**	
	**EC_50_ (μg/mL)^a^**	**EC_50_ (μg GAE/mL)^b^**	**FRAP (mmol Fe^2+^/g)^c^**	**FRAP (mmol Fe^2+^/g GAE)^d^**	**IC_50_ (μg/mL)^e^**	**IC_50_ (μg GAE/mL)^d^^,^^f^**
MED	15.46 ± 0.38^F^	3.19	4.40 ± 0.10^B^	21.35	20.02 ± 1.95^F^	4.13
DEF	6.91 ± 0.22^D^	3.42	9.46 ± 0.04^D, E^	19.12	6.87 ± 0.59^C, D^	3.40
EAF	6.04 ± 0.07^D^	3.53	9.02 ± 0.11^D^	15.44	5.81 ± 0.40^B, C^	3.40
BF	11.79 ± 0.50^E^	3.50	6.15 ± 0.23^C^	20.75	11.34 ± 0.76^E^	3.36
WR	51.32 ± 0.93^G^	3.32	1.31 ± 0.04^A^	20.33	49.73 ± 2.05^G^	3.21
QU	1.63 ± 0.07^A^	–	36.02 ± 1.1^H^	–	1.85 ± 0.12^A^	–
TX	4.34 ± 0.22^C^	–	10.83 ± 0.32^E^	–	8.47 ± 0.45^C, D, E^	–
BHA	2.90 ± 0.14^B^	–	16.13 ± 0.83^F^	–	3.16 ± 0.22^A, B^	–
BHT	6.54 ± 0.28^D^	–	18.89 ± 0.42^G^	–	9.31 ± 0.16^D, E^	–

*Results are presented as mean values (± SD for replicates, n = 3) calculated per dry weight of the extract or positive control (QU, quercetin; TX, Trolox; BHA; BHT). For extract codes see Table 1. Different superscripts (capitals) in each column indicate significant differences in the means at p < 0.05*.

^a, b^Scavenging efficiency (amount of antioxidant needed to decrease the initial DPPH concentration by 50%) expressed as follows:

a in μg of the dry extract or standard/mL of the DPPH solution;

b *in μg of phenolics/mL of the DPPH solution (values obtained by converting the original EC_50_ values using the TPC levels)*.

c *Values expressed per g of the dry extract or standard*.

d *Values expressed per g of the phenolics (obtained by converting the original FRAP values using the TPC levels)*.

^e, f^Linoleic Acid (LA) Peroxidation test; inhibition concentration (amount of antioxidant needed to decrease the LA-peroxidation by 50%) expressed as follows:

e in μg of the dry extract or standard/mL of the LA solution;

f *in μg of phenolics/mL of the LA solution (values obtained by converting the original IC_50_ values using the TPC levels)*.

**Table 4:** Correlation coefficients (*r*) and probability (*p*) values of linear relationships between antioxidant activity parameters and phenolic contents.

***r (p)* for:**	**Antioxidant activity (chemical models)**			**Antioxidant activity (human plasma model)**			
	**DPPH**	**FRAP**	**TBARS**	**3NT-Fg**	**TBARS**	**FOX-1**	**FRAP**
TPC	−0.8277 (0.084)	0.9721 (0.006)^**^	−0.8696 (0.055)	0.5094 (0.052)	0.8657 (0.000)^***^	0.7567 (0.001)^***^	0.9877 (0.000)^***^
TFC	−0.8122 (0.095)	0.9606 (0.009)^**^	−0.8536 (0.066)	0.4733 (0.075)	0.7335 (0.002)^**^	0.6273 (0.012)^*^	0.9284 (0.000)^***^
TPA	−0.7273 (0.164)	0.7586 (0.137)	−0.7453 (0.148)	0.5084 (0.053)	0.9018 (0.000)^***^	0.8001 (0.000)^***^	0.9535 (0.000)^***^
TAC	−0.1415 (0.820)	−0.1517 (0.808)	−0.1417 (0.820)	0.2173 (0.437)	0.4021 (0.134)	0.2125 (0.447)	0.5691 (0.021)^*^

**Figure 2:**
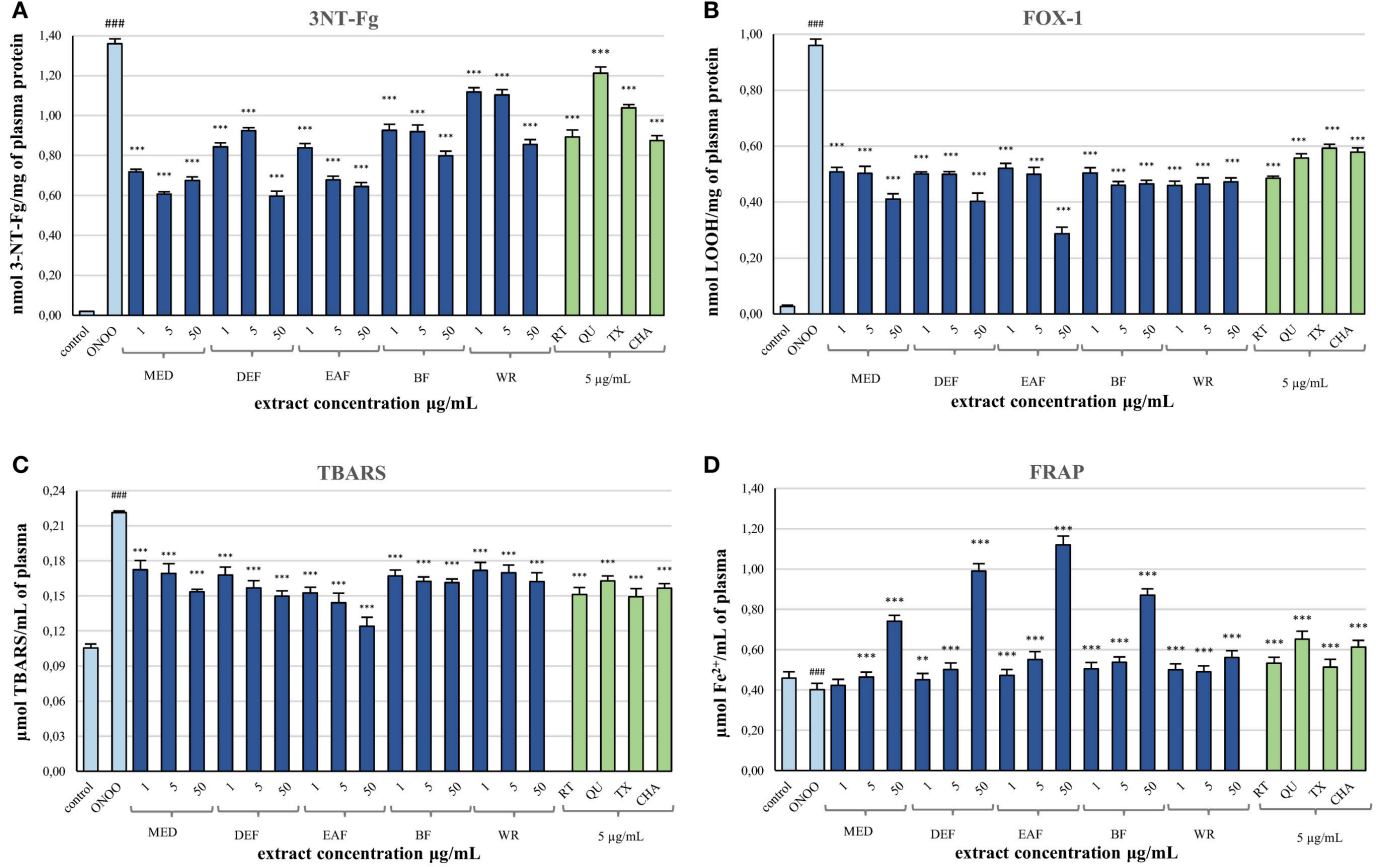
Effects of *P. spinosa* flower dry extracts on human plasma exposed to oxidative stress: **(A)** effects on the nitration of tyrosine residues in plasma proteins and formation of 3-nirotyrosine, 3-NT-Fg; effects on the peroxidation of plasma lipids including formation of lipid hydroperoxides, LOOH **(B)**, and thiobarbituric acid-reactive substances, TBARS **(C)**; **(D)** effects on ferric reducing ability of plasma, FRAP. Results are presented as means ± SE (*n* = 10) for repeated measures: ^###^*p* < 0.001 for control plasma vs. ONOO^−^-treated plasma (without the extracts); ^**^p < 0.01, ^***^p < 0.001 for ONOO^−^-treated plasma in the presence of the extracts (1, 5, or 50 μg/mL) or standards (5 μg/mL) vs. ONOO^−^-treated plasma in the absence of the extracts. Standards: RT, rutin; QU, quercetin; TX, Trolox; CHA, chlorogenic acid.

Activity and concentration parameters according to Tables 2, 3, and Figure 2. Asterisks mean statistical significance of the estimated linear relationships (

* *p < 0.05*,

** *p < 0.01*,

*** *p < 0.001)*.

### Protective effects on human plasma components

After demonstrating the promising antioxidant capacity in chemical assays, the extracts were examined in terms of their effects on human plasma exposed to oxidative stress induced by peroxynitrite (ONOO^−^; 100 μM). In comparison to the control (untreated) samples, the peroxynitrite-stimulated plasma exhibited a considerably enhanced level (Figure 2A; *p* < 0.001) of 3-nitrotyrosine in plasma proteins (3-NT-Fg, a marker of protein nitration), a strong increase of lipid hydroperoxides (ferric-xylenol orange assay, FOX-1) and thiobarbituric acid-reactive substances (TBARS)—markers of lipid peroxidation (Figures 2B,C; *p* < 0.001), as well as a noticeable decrease (Figure 2D; *p* < 0.001) in ferric reducing ability (FRAP, a marker of total antioxidant status of plasma). In plasma samples incubated with ONOO^−^ in the presence of the extracts (at 1–50 μg/mL), the rate of oxidative/nitrative damage was significantly reduced (Figures 2A–C; *p* < 0.05). All tested extracts effectively diminished the nitration of tyrosine residues (Figure 2A; *p* < 0.001)—by about 15–50% and 36–54% at 1 and 50 μg/mL, respectively. The impact of extraction solvent on the anti-nitrative activity was less pronounced than that observed in chemical tests; still, WR was again the least effective at each concentration level (*p* < 0.05). Among the other extracts, MED displayed the strongest activity (50–58% decrease in tyrosine nitration); however, only the effects of EAF, BF, and WR were dose-dependent (Figure 2A; *p* < 0.05). In consequence, the correlation between the percentage inhibition of tyrosine nitration and the phenolic contents (TPC, TFC, TPA, TAC) was not significant (Table 4). All of the tested extracts exhibited also protective properties against peroxynitrite-induced lipid peroxidation (Figures 2B,**C**; *p* < 0.001), regardless of the concentration level. In samples pre-incubated with the medium extracts concentration (5 μg/mL), the levels of hydroperoxides and TBARS decreased by about 47–53% and 21–34%, respectively. The solvent effects were negligible, although EAF at 50 μg/mL was the most effective antioxidant, able to reduce the peroxidation parameters by about 70% in FOX-1 and 45% in TBARS tests. The inhibitory activity on lipid peroxidation was dose-dependent for all extracts if measured by TBARS (Figure 2C; *p* < 0.05); however, in the FOX-1 assay only some dose-effects for MED, DEF and EAF were significant, i.e., differences observed between the two lowest concentration levels (1–5 μg/mL) and the highest dose of 50 μg/mL (Figure 2B; *p* < 0.05). Consequently, relatively strong correlations (*|r|* > 0.73, *p* < 0.01) were observed between the percentage of lipid peroxidation assayed by TBARS and the phenolic contents (TPC, TFC, TPA), whereas the analog relationships for FOX-1 test were weaker (*|r|* > 0.62, *p* < 0.01) (Table 4). All of the extracts were also able to normalize and/or enhance the total antioxidant status of ONOO^−^-treated plasma (FRAP), depending on the concentration used, and only MED at 1 μg/mL did not change significantly the FRAP value of the oxidized plasma (Figure 2D; *p* > 0.05). For all other extracts, a significant improvement in the plasma reducing ability was observed with the increase up to about 190% vs. the ONOO^−^-stimulated plasma and 140% vs. the control sample for EAF at 50 μg/mL (Figure 2D; *p* < 0.001). Strong dose-dependency of the analytical response was found, and thus strong relationships (*|r|* > 0.92, *p* < 0.001) between the percentage increase in the FRAP values of the oxidized plasma and the TPC, TFC, and TPA levels (Table 4).

Simultaneously, the ONOO^−^-treated plasma was incubated with 5 μg/mL of standard phenolics (Figures 2A–D). In contrast to results of the chemical tests, in the plasma model the pure compounds were merely comparable or inferior to the blackthorn extracts in terms of the antioxidant activity. For instance, the range of percentage inhibition of tyrosine nitration achieved for the standards (ca. 11–34%) was lower than for the extracts (ca. 19–58%) applied at the same concentration of 5 μg/mL (Figure 2A). Moreover, the activity of MED and EAF at 5 μg/mL was significantly higher (*p* < 0.001) than that of all standards. Furthermore, quercetin—the strongest antioxidant in chemical models—counteracted the plasma nitration significantly less effectively than all extracts (Figure 2A; *p* < 0.001). Similar trend was observed for ONOO^−^-induced lipid peroxidation, both with FOX-1 detection—only rutin exhibited protective activity comparable to that of the extracts at 5 μg/mL (Figure 2B; *p* > 0.05), and with TBARS test—overlapped ranges of percentage inhibition of peroxidation were found for the standards (ca. 26–32%) and the extracts (21–34%) at 5 μg/mL (Figure 2C; *p* > 0.05). Only in the FRAP assay, two of the standards—quercetin and chlorogenic acid—enhanced the reducing ability of plasma more strongly than the extracts at the corresponding concentration (Figure 2D; *p* < 0.001).

### Inhibitory effects on pro-inflammatory enzymes

The extracts inhibited the activity of lipoxygenase (LOX) and hyaluronidase (HYAL) in a dose-dependent manner, but with different responses toward particular enzyme (Table 5). Considering the IC_50_ values expressed in μg/U, the extracts were stronger inhibitors of LOX than HYAL. In both tests the strongest effects observed for EAF and DEF were intermediate between those of phenolic standards and indomethacin, a strong nonsteroidal anti-inflammatory drug. The inhibitory activity toward both enzymes was strongly correlated with the TPC levels (*|r|* > 0.88, *p* < 0.05). The responses in the LOX test were also strongly TFC-dependent (*r* = −0.8868, *p* < 0.05), while in the HYAL assay this relationship was noticeable (*r* = −0.8691) but not significant (*p* = 0.056).

**Table 5:** Inhibitory activity of *P. spinosa* flower dry extracts on lipoxygenase (LOX) and hyaluronidase (HYAL).

**Analyte**	**LOX**		**HYAL**	
	**IC_50_ (μg/mL)^a^**	**IC_50_ (μg/U)^b^**	**IC_50_ (μg/mL)^a^**	**IC_50_ (μg/U)^b^**
MED	327.36 ± 5.93^F^	7.85	51.74 ± 2.16^E^	23.00
DEF	150.36 ± 4.47^C^	3.60	21.40 ± 0.76^B^	9.51
EAF	135.36 ± 5.55^B^	3.25	21.27 ± 0.16^B^	9.45
BF	171.10 ± 1.36^E^	4.11	42.23 ± 0.99^D^	18.77
WR	479.50 ± 3.38^G^	11.50	121.72 ± 5.73^F^	54.10
QU	89.23 ± 2.13^A^	2.14	30.78 ± 1.84^C^	13.65
RT	162.70 ± 3.70^D^	3.90	54.63 ± 2.61^E^	24.23
CHA	166.83 ± 7.15^D, E^	4.00	28.59 ± 1.21^C^	12.68
IND	92.60 ± 3.71^A^	2.22	12.77 ± 0.91^A^	5.66

*Results are presented as mean values ± SD (n = 3) calculated per dry weight of the extract or positive control (QU, quercetin; RT, rutin; CHA, chlorogenic acid; IND, indomethacin). For extract codes see Table 1. Different superscripts (capitals) in each column indicate significant differences in the means at p < 0.05*.

^a, b^Inhibition concentration (amount of analyte needed for 50% inhibition of enzyme activity) expressed as follows:

a in μg of the dry extract or standard/mL of the enzyme solution;

b *in μg of the extracts/enzyme unit (U)*.

### Influence on cells viability

**Figure 3:**
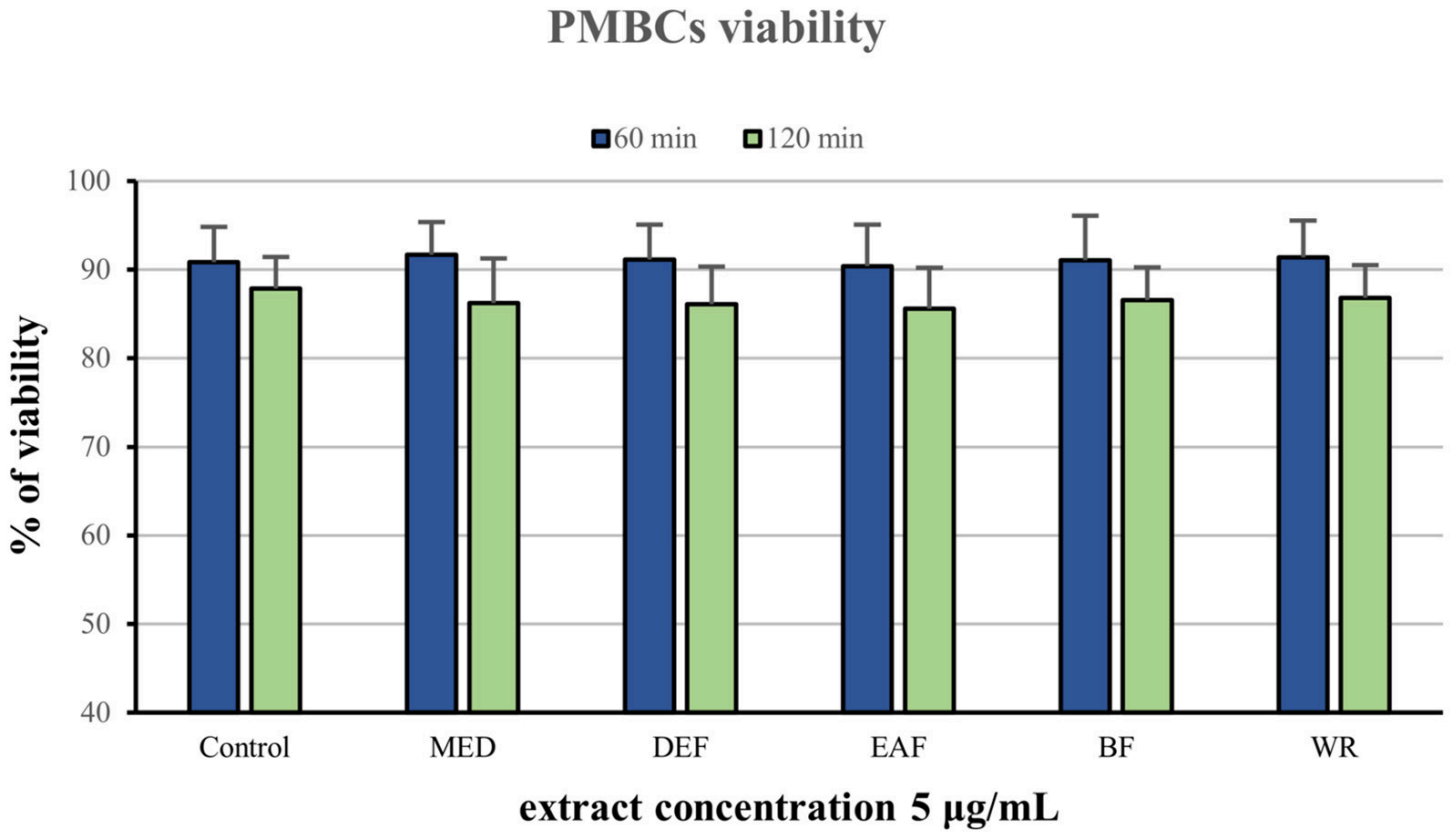
Viability of peripheral blood mononuclear cells (PMBCs) after 60 and 120 min of incubation with *P. spinosa* flower dry extracts at 5 μg/mL.

The potential cytotoxicity of the extracts was evaluated in a model of PBMCs after 60 and 120 min of incubation with the extracts at 5 μg/mL. Cellular safety of the extracts was evidenced by the lack of significant differences (*p* > 0.05) in cell viability observed between PBMCs incubated with the extracts (85.6–90.5% viability) and the control (untreated) samples (87.85–90.85 viability), regardless of the incubation time and extraction solvent (Figure 3).

## Discussion

As beneficial for human health, including prevention of CVD, polyphenol-rich extracts are widely used in the preparation of dietary supplements and pharmaceuticals (Franz et al., 2011). Extraction from natural matrices is thus the crucial step in their utilization as it affects extract composition, activity, and yield. Generally, alcohol and alcohol-water mixtures are the best extractants for low-molecular-weight polyphenols of high antioxidant potential and good bioavailability (Manach et al., 2005). The crude extracts are often fractionated to remove fat-soluble ballast substances (chlorophylls and waxes) and thereby purify and concentrate the phenolic fractions (Rana et al., 2016). The results from the present study confirmed these observations: the defatted methanol-water (7:3, v/v) extract from the blackthorn flowers (MED), demonstrated to be a rich source of phenolic compounds, could be further enriched by liquid-liquid partitioning with organic solvents (Table 2). The TPC in the richest fractions EAF and DEF, as determined by the Folin-Ciocalteu (FC) assay, were comparable to those found in the extracts considered important in CVD prevention, for instance in commercial ethanol extracts of grape seed (ca. 630–670 mg GAE/g dw) (Baydara et al., 2004) or in ethyl acetate fractions of green tea and green mate (ca. 480–580 mg GAE/g dw) (Erol et al., 2009). The previous literature data referring to *P. spinosa* are limited to studies on fruits, branches, and leaves. According to Pinacho et al. (2015), the highest TPC level quantified by the Prussian Blue method (PB) was found for ethanol fraction from branches (732.34 mg GAE/g dw), followed by those from fruits (359.11 mg GAE/g dw) and leaves (228.56 mg GAE/g dw). Despite some differences in chemistry of the PB assay in comparison to the standardized FC test, in the preliminary studies focused on selection of the plant material for the present work, we have observed the magnitude of phenolic levels to some extent similar, which enables ordering of the blackthorn tissues in respect to the TPC values as follows: branches > flowers > fruits ≥ leaves. Therefore, the flowers were found promising for further studies.

The present paper is the first presenting comprehensive LC-MS profile of *P. spinosa* flowers (Figure 1, Table 1). The previous studies aimed at isolation and resulted in structure elucidation of 16 flavonoids (Sakar and Kolodziej, 1993; Olszewska and Wolbiś, 2001, 2002a) and five proanthocyanidins (Kolodziej et al., 1991), the presence of which was now confirmed in the flower dry extracts. In addition, 36 constituents, mainly flavonoids, and phenolic acids, were reported here for the first time for the blackthorn flowers (Table 1). With nearly 60 components detected, in contrast to only 25 observed in the leaves (Owczarek et al., 2017), 26 in branches (Pinacho et al., 2015), and 29 in fruits (Guimarães et al., 2013), the phenolic matrix of the flowers is obviously the most complex. A distinctive qualitative feature of the flower extracts is a vast diversity of the flavonoid fraction (thirty seven peaks). While according to Owczarek et al. (2017) flavonoids prevail also in the leaf samples, only 14 structures have been detected in this organ. The branches and fruits of *P. spinosa* may be in turn distinguished by the presence of numerous proanthocyanidins (twelve peaks in branches) and anthocyanins (eight peaks in fruits), respectively (Guimarães et al., 2013; Pinacho et al., 2015). In phytotherapy, the blackthorn flowers are indeed recognized as a flavonoid herbal product (Hoppe, 1981; Poonam et al., 2011). High content of flavonoids found in the present study and their prevalence in flower dry extracts (Table 2), were in agreement with these findings. In addition to flavonoids, the analyzed extracts contained moderate levels of proanthocyanidins and phenolic acids (Table 2). The TPA level of MED is in accordance with the results reported recently by Ropiak et al. (2016) for aqueous acetone extracts of blackthorn flowers (40 mg/g dw). As indicated in the latter paper, the average degree of polymerization of flower procyanidins is 2.9, which means that the majority of TPA fraction is within the bioavailable range of molecular masses (Manach et al., 2005). Thus, the composition of the flower extracts of blackthorn appears promising in the context of CVD and potential industrial application. Regular intake of flavonoids with the diet or supplements is associated with reduced risk of cardiovascular episodes and mortality (Alissa and Ferns, 2012). In *in vivo* studies, they have been shown to improve antioxidant status, exert anti-atherosclerotic and anti-atherothrombotic effects in early stages of atherosclerosis development (e.g., decrease LDL oxidation), modulate lipid metabolism (e.g., normalize LDL/HDL profile), improve capillary permeability (vasoprotective effects) and endothelial function, and increase nitric oxide release (vasodilatory effects) (Middleton et al., 2000; Alissa and Ferns, 2012). As the most common group of polyphenols in the human diet, flavonoids are also regarded as safe in internal applications. Like flavonoids, both low-molecular-mass proanthocyanidins and phenolic acids have been shown to modulate lipid metabolism, increase plasma antioxidant capacity, improve vascular functions, and reduce platelet activity in humans (Manach et al., 2005).

As antioxidant activity of polyphenols is one of the most important aspects of their beneficial effects in CVD (Middleton et al., 2000; Alissa and Ferns, 2012; Quiñones et al., 2013; Santilli et al., 2015) we decided to verify it in complementary chemical and biological models that reflect various direct and complex mechanisms. Although simple chemical *in vitro* assays are hardly relevant to *in vivo* conditions, they enable preliminary screening for potential mechanisms and comparison with the literature data. In the present study, we employed three the most frequently used tests of both single electron transfer (DPPH, FRAP) and hydrogen atom transfer basic mechanisms (TBARS) to explore direct interactions of the extracts with free-radicals and transition metal ions as well as to study some of these effects in a model of lipid peroxidation. The results demonstrated that the flower extracts are potent and universal antioxidants, and that their dose-dependent activity is determined by the contents of phenolics, primarily flavonoids, and strongly influenced by the fractionation solvent (Tables 3, 4). Also the extracts produced from other organs of *P. spinosa* have recently been reported effective DPPH scavengers, as well as inhibitors of lipid peroxidation (Guimarães et al., 2014; Pinacho et al., 2015). Comparing our results to those of Pinacho et al. (2015) and in terms of capacity of BHA after 60 min of incubation, it seems that the activity of flower extracts (EAF, DEF) is comparable to that of branches, and superior to those of leaves and fruits. Moreover, the comparison of our results from TBARS assay and the results obtained by Guimarães et al. (2014) in terms of activity of Trolox, shows that the lipid peroxidation inhibitory activity of flower extracts (EAF, DEF) is higher than that of phenolic-enriched extracts from wild fruits. On the other hand, the outcomes from DPPH test suggest similar scavenging activity for flower and fruit extracts.

To give a more accurate approximation of the possible *in vivo* effects of the flower extracts, we extended the *in vitro* study to the model of human plasma exposed to oxidative stress. The stress conditions were induced by peroxynitrite (ONOO^−^), a powerful oxidative and nitrative species, generated *in vivo* by the reaction of nitric oxide (NO) and superoxide anion (${\text{O}}_{2}^{•-}$), and involved in the pathophysiology of various inflammatory, neurodegenerative, and especially cardiovascular disorders including atherosclerosis, myocardial infarction and chronic heart failure (Ronson et al., 1999). The concentration of ONOO^−^ used in our *in vitro* study (100 μM) enables quantitative measurements of the changes induced in plasma components and corresponds to its levels *in vivo*, that can be reached in local compartments in the conditions of accelerated production of NO and ${\text{O}}_{2}^{•-}$, e.g., during a serious inflammation of blood vessels, when its synthesis can increase up to 50–100 μM/min (Szabo et al., 2007). During the study, the plasma samples were pre-incubated with the extracts at the levels of 1–50 μg/mL, equivalent to 0.06–29.2 μg GAE/mL, depending on the extract. From the physiological point of view, the concentrations of phenolic substances that are likely to occur in blood plasma *in vivo* after oral supplementation can reach up to 5–7 μM, depending on the food matrix (Manach et al., 2005). For example, it has been reported that 100-mg dose of quercetin from onions and 150-mg dose of pure isoquercitrin (quercetin-3-glucoside) resulted in the concentrations up to 7.6 and 5 μM in plasma, respectively, which is an equivalent of 1.5–2.3 μg/mL of quercetin (Hollmann, 2004; Manach et al., 2005). It has also been suggested that the bioavailability of kaempferol and its glycosides, the dominant flavonoids of blackthorn flowers according to our results, is higher than that of quercetin (DuPont et al., 2004). Therefore, the lower levels of the extracts (1–5 μg/mL) used in the study appear to closely correspond to the range of physiological level of plant-derived phenolic compounds available after oral administration. In accordance with the common practice of *in vitro* studies (Kolodziejczyk et al., 2011; Kolodziejczyk-Czepas et al., 2013, 2014), the extracts were studied also at 50 μg/mL to enable that of observation of all possible effects of their interaction with peroxynitrite.

The results from biological model confirmed our hypothesis that antioxidant activity of the *P. spinosa* flower extracts may be crucial in understanding their beneficial effects in CVD *in vivo*. The analyzed extracts not only enhanced the total antioxidant status of the ONOO^−^-treated plasma but also effectively reduced the levels of well-known oxidative stress biomarkers—products of protein nitration (3-nitrotyrosine) and lipid peroxidation (hydroperoxides and TBARS) (Figure 3). In CVD patients, the increased levels of these biomarkers are good predictors of cardiovascular events and are correlated with prothrombotic, proatherogenic, and pro-inflammatory intravascular effects, plaque instability, and endothelial dysfunctions (Lee et al., 2012; Thomson, 2015). The mechanism of action of the blackthorn extracts in the protection of plasma proteins and lipids probably involves a direct scavenging of peroxynitrite or/and secondary radicals formed in the induced chain reactions. It is consistent with the noticeable scavenging potential of the extracts toward the model radical (DPPH), and with the accumulating evidence of anti-radical effects of polyphenols against ONOO^−^ and ONOO^−^-related radicals operating in plasma, such as ${\text{O}}_{2}^{•-}$, NO, ^•^NO_2_, ^•^OH, etc. (Heijnen et al., 2001). Despite some arguments being presented for the impact of blackthorn polyphenols on the target activity in plasma, the differences between the examined extracts were not as evident as in the case of chemical tests. The discrepancies may be an effect of complexity and ontogenetic diversity of blood plasma, as well as differences in the sensitivity to ONOO^−^ between the plasma samples. Some interactions between endogenous plasma constituents and blackthorn phenolics could also be the reason. On the other hand, the complexity of the extracts and synergic effects of their individual components are probably behind the superior activity profile revealed by the extracts comparing to the pure standards. Finally, it is of note that all beneficial effects of the extracts on plasma were measurable at physiological concentrations (1–5 μg/mL), which do not deteriorate the viability of PBMCs and may be regarded as safe.

Pathogenesis of CVD covers, apart from oxidative stress, also a closely related process of inflammation (Biswas, 2016). To evaluate the potential anti-inflammatory effects of the blackthorn extracts, we decided to study their impact on two model pro-inflammatory enzymes. The first belongs to the family of lipoxygenases, enzymes catalyzing dioxygenation of polyenoic fatty acids in biological membranes and lipoproteins, and producing key chemokines and ROS, such as leukotrienes and ${\text{O}}_{2}^{•-}$, associated with the development of oxidative stress-related inflammatory pathologies of CVD, e.g., myocardial infarction/reperfusion injury (Schneider and Bucar, 2005). The second one—hyaluronidase—is a spreading factor increasing tissues (i.a. vascular) permeability during the inflammatory processes by degrading hyaluronan, an anti-inflammatory extracellular matrix component (Girish et al., 2009). Both enzymes are targets of many synthetic drugs used in anti-inflammatory therapies, but it has been shown that plant extracts, especially those containing phenolics, can also significantly inhibit their activity (Schneider and Bucar, 2005; Piwowarski et al., 2011). Indeed, the investigated extracts, rich in polyphenols, were found to be inhibitors of both enzymes (Table 5). The activity of the most active fractions was between those of the commercial agents—indomethacin (nonsteroidal anti-inflammatory drug) and rutin (flavonoid vasoprotective agent), while the basic methanol-water extract was slightly less active. However, with the results of antioxidant activity tests, these findings suggest that the extracts might influence both interdependent processes of oxidative stress and inflammation.

## Conclusions

This work is the first comprehensive phytochemical and activity study of the flower extracts of *P. spinosa*. The detailed LC-MS patterns presented here can be recommended as reference fingerprints useful in authentication and standardization studies of blackthorn flowers and flower extracts. Distinct phenolic profiles, significant antioxidant effects comparable to or surpassing the activity of pure phenolics in both chemical and biological models, noticeable inhibitory effects on pro-inflammatory enzymes, and cellular safety suggest that the investigated extracts may be promising candidates for the production of pharma- and nutraceuticals effective in cardiovascular protection. Considering both the yield and the activity, the defatted methanol-water (7:3, v/v) extract and its diethyl ether and ethyl acetate fractions appear to be the most advantageous for biological applications. As the observed capacities might be considered as some of the mechanisms behind the activity of blackthorn phenolics within the circulatory system, the present study forms a basis for their further studies in the context of CVD. For instance, the demonstrated protective effects of the extracts against ONOO^−^-induced changes in plasma components and ability to inhibit pro-inflammatory enzymes might be cross-linked with their possible anti-atherogenic, anticoagulant, and antiplatelet functions or ability to influence endothelium, which should be addressed in future research.

## Author contributions

AM, AO, and MO planned the experiments and wrote the manuscript. AM, AO, MM, AP, and JK-C performed the experiments. AM and AO carried out data analysis. PN acted as the consultant on biological methods. MO supervised all work. All authors critically revised and approved the final version of the manuscript.

### Conflict of interest statement

The authors declare that the research was conducted in the absence of any commercial or financial relationships that could be construed as a potential conflict of interest.
